# Venous thromboembolism and infection

**DOI:** 10.7196/AJTCCM.2021.v27i3.171

**Published:** 2021-10-04

**Authors:** S D Maasdorp

**Affiliations:** Department of Internal Medicine, Faculty of Health Sciences, University of the Free State, Bloemfontein, South Africa

## Editorial

Venous thromboembolism (VTE), manifesting as either deep venous thrombosis (DVT), pulmonary embolism (PE) or both, remains a major cause of cardiovascular morbidity and mortality worldwide. The incidence of VTE ranges from 79 - 269 per 100 000 people in a population, and is almost eight times higher after the age of 80 years than below the age of 50 years.^[1]^ In the USA, the annual death rate associated with VTE is 60 000 - 100 000, but may be as high as 300 000.^[1]^ Notably, one of the first manifestations of acute PE can be sudden death in up to 25% of cases. ^[2]^ Long-term complications of VTE include post-thrombotic syndrome and chronic thromboembolic pulmonary hypertension.^[3]^ Virchow’s triad is often used to describe the underlying pathophysiological mechanisms by which venous thrombi are formed. The triad consists of stasis of venous blood flow, hypercoagulability, and damage to the blood vessel wall.^[4]^ Various risk factors predispose to VTE. Classic examples include prolonged immobilisation, use of oral contraceptives, orthopaedic surgery and cancer.^[5]^ Infections have long been known to predispose to VTE.^[6]^ The most dramatic example is the recent COVID-19 pandemic caused by SARS-CoV-2. COVID-19 first broke out in China in December 2019 and thereafter rapidly spread across the globe. By the end of July 2021, the world had recorded ~200 million cases and more than 4.2 million deaths.^[7]^ At the same time, South Africa (SA) had 2.45 million cases and more than 72 000 deaths.^[7]^ A large proportion of COVID-19 patients (~30%) admitted to the intensive care unit developed VTE.^[8]^ The underlying mechanisms for the elevated risk for thrombus in patients with COVID-19 could include immune-mediated thrombotic mechanisms, complement activation, macrophage activation syndrome, antiphospholipid antibody syndrome, hyperferritinaemia, and renin-angiotensin system dysregulation.^[9]^ Less dramatic than COVID-19, but not less important, are the ongoing pandemics of HIV/AIDS and tuberculosis (TB). There are ~38 million people living with HIV worldwide^[10]^ and ~7.5 million of them live in SA.^[11]^ There were 360 000 cases of TB and 58 000 deaths reported in SA in 2019.^[12]^ Both HIV and TB are risk factors for thrombosis. The increased risk for thrombus formation associated with HIV include an increase in procoagulant factors such as tissue factor, lupus anticoagulant and homocysteine, a reduction in anticoagulant factors such as antithrombin, protein S and C, and endothelial dysfunction as a result of chronic systemic inflammation.^[13–14]^ A recent population-based study found an adjusted hazard ratio of 1.4 for VTE associated with HIV, indicating an increased risk for VTE in HIV-infected individuals.^[15]^ Tuberculosis, another condition that leads to chronic inflammation, has similarly been associated with a high risk for thrombosis.^[16]^ A recent meta-analysis by Danwang *et al*.^[16]^ reported that VTE has a prevalence of 3.5% in patients with active TB with an odds ratio of 2.9.^[16]^ In this issue of the AJTCCM, Moodley *et al*.^[17]^ eloquently refocus our attention on VTE associated with HIV and TB. The authors compared clinical characteristics of hospitalised patients with VTE, stratified by HIV and TB status. One hundred patients were admitted to hospital with VTE during the study period of 9 months, representing 1.5% of all patients admitted during that time. The investigators found a high prevalence of HIV (59%) and TB (39%) in the study cohort. Most of the HIV-infected patients (74.6%) were initiated onto antiretroviral therapy prior to being diagnosed with VTE. This may reflect a possible link between immune reconstitution and an increased risk of thrombosis during the initial period of antiretroviral treatment. The study by Moodley *et al*. again confirms the high prevalence of HIV and TB in patients with VTE in SA, similar to previously published studies.^[17]^ Moreover, we are starkly reminded that in SA, we are in reality dealing with a triple pandemic of HIV, TB and now COVID-19, all of which predispose patients to thrombus formation and VTE.^[18]^ We therefore need to remain vigilant and maintain a high index of suspicion for VTE in patients with infectious diseases admitted to our healthcare facilities.
